# Supplementary material for: Resolving the full spectrum of human genome variation using Linked-Reads
Source: Genome Res. 2019 Apr;29(4):635–45. doi: 10.1101/gr.234443.118 (PMC6442396; doi:10.1101/gr.234443.118)
Supplement: Supplemental Material [file supp_gr.234443.118_Supplemental_longranger-2.2.zip › longranger-2.2.tar.bz2!/lr2.2-public/lib/stan/cmdstan/stan/doc/api/html/math_2ode_2coupled__ode__system_8hpp_source.html]

Stan: src/stan/math/ode/coupled\_ode\_system.hpp Source File


|  |  |
| --- | --- |
|  | Stan  2.5.0  probability, sampling & optimization |


- Main Page
- Related Pages
- Namespaces
- Classes
- Files

- File List
- File Members

All Classes Namespaces Files Functions Variables Typedefs Enumerations Enumerator Friends Macros Pages

- src
- stan
- math
- ode

coupled\_ode\_system.hpp

Go to the documentation of this file.

1 #ifndef STAN\_\_MATH\_\_ODE\_\_COUPLED\_ODE\_SYSTEM\_HPP

2 #define STAN\_\_MATH\_\_ODE\_\_COUPLED\_ODE\_SYSTEM\_HPP

3

4 #include <ostream>

5 #include <vector>

6

7 #include <stan/math/error\_handling/matrix/check\_matching\_sizes.hpp>

8

9 namespace stan {

10

11  namespace math {

12

26  template <typename F, typename T1, typename T2>

27  struct coupled\_ode\_system {

28  };

29

30

41  template <typename F>

42  struct coupled\_ode\_system<F, double, double> {

43

44  const F& f\_;

45  const std::vector<double>& y0\_dbl\_;

46  const std::vector<double>& theta\_dbl\_;

47  const std::vector<double>& x\_;

48  const std::vector<int>& x\_int\_;

49  const int N\_;

50  const int M\_;

51  const int size\_;

52  std::ostream\* msgs\_;

53

66  coupled\_ode\_system(const F& f,

67  const std::vector<double>& y0,

68  const std::vector<double>& theta,

69  const std::vector<double>& x,

70  const std::vector<int>& x\_int,

71  std::ostream\* msgs)

72  : f\_(f),

73  y0\_dbl\_(y0),

74  theta\_dbl\_(theta),

75  x\_(x),

76  x\_int\_(x\_int),

77  N\_(y0.size()),

78  M\_(theta.size()),

79  size\_(N\_),

80  msgs\_(msgs) {

81  }

82

98  void operator()(const std::vector<double>& y,

99  std::vector<double>& dy\_dt,

100  double t) {

101  dy\_dt = f\_(t,y,theta\_dbl\_,x\_,x\_int\_,msgs\_);

102  stan::math::check\_matching\_sizes("coupled\_ode\_system(%1%)",

103  y,"y",dy\_dt,"dy\_dt",

104  static\_cast<double\*>(0));

105  }

106

112  int size() const {

113  return size\_;

114  }

115

128  std::vector<double> initial\_state() {

129  std::vector<double> state(size\_, 0.0);

130  for (int n = 0; n < N\_; n++)

131  state[n] = y0\_dbl\_[n];

132  return state;

133  }

134

145  std::vector<std::vector<double> >

146  decouple\_states(const std::vector<std::vector<double> >& y) {

147  return y;

148  }

149

150  };

151

152  }

153

154 }

155

156 #endif

N\_

int N\_

**Definition:** mdivide\_left.hpp:20

stan::math::coupled\_ode\_system< F, double, double >::operator()

void operator()(const std::vector< double > &y, std::vector< double > &dy\_dt, double t)

Calculates the derivative of the coupled ode system with respect to the specified state at the specif...

**Definition:** coupled\_ode\_system.hpp:98

stan::math::coupled\_ode\_system< F, double, double >::x\_

const std::vector< double > & x\_

**Definition:** coupled\_ode\_system.hpp:47

stan::math::coupled\_ode\_system< F, double, double >::msgs\_

std::ostream \* msgs\_

**Definition:** coupled\_ode\_system.hpp:52

stan::math::coupled\_ode\_system< F, double, double >::x\_int\_

const std::vector< int > & x\_int\_

**Definition:** coupled\_ode\_system.hpp:48

stan::math::coupled\_ode\_system< F, double, double >::N\_

const int N\_

**Definition:** coupled\_ode\_system.hpp:49

stan::math::coupled\_ode\_system< F, double, double >::y0\_dbl\_

const std::vector< double > & y0\_dbl\_

**Definition:** coupled\_ode\_system.hpp:45

stan::math::coupled\_ode\_system< F, double, double >::theta\_dbl\_

const std::vector< double > & theta\_dbl\_

**Definition:** coupled\_ode\_system.hpp:46

check\_matching\_sizes.hpp

stan::math::coupled\_ode\_system< F, double, double >::M\_

const int M\_

**Definition:** coupled\_ode\_system.hpp:50

stan::math::coupled\_ode\_system< F, double, double >::initial\_state

std::vector< double > initial\_state()

Returns the initial state of the coupled system, which is identical to the base ODE original state in...

**Definition:** coupled\_ode\_system.hpp:128

stan::math::coupled\_ode\_system< F, double, double >::size

int size() const

Returns the size of the coupled system.

**Definition:** coupled\_ode\_system.hpp:112

stan::math::coupled\_ode\_system< F, double, double >::coupled\_ode\_system

coupled\_ode\_system(const F &f, const std::vector< double > &y0, const std::vector< double > &theta, const std::vector< double > &x, const std::vector< int > &x\_int, std::ostream \*msgs)

Construct the coupled ODE system from the base system function, initial state, parameters, data and a stream for messages.

**Definition:** coupled\_ode\_system.hpp:66

stan::math::coupled\_ode\_system< F, double, double >::size\_

const int size\_

**Definition:** coupled\_ode\_system.hpp:51

stan::math::coupled\_ode\_system< F, double, double >::decouple\_states

std::vector< std::vector< double > > decouple\_states(const std::vector< std::vector< double > > &y)

Returns the base portion of the coupled state.

**Definition:** coupled\_ode\_system.hpp:146

stan::math::coupled\_ode\_system< F, double, double >::f\_

const F & f\_

**Definition:** coupled\_ode\_system.hpp:44

stan::math::size

int size(const std::vector< T > &x)

**Definition:** size.hpp:11

size\_

size\_t size\_

**Definition:** dot\_self.hpp:18

stan::math::coupled\_ode\_system

Base template class for a coupled ordinary differential equation system, which adds sensitivities to ...

**Definition:** coupled\_ode\_system.hpp:27

stan::math::check\_matching\_sizes

bool check\_matching\_sizes(const char \*function, const T\_y1 &y1, const char \*name1, const T\_y2 &y2, const char \*name2, T\_result \*result)

**Definition:** check\_matching\_sizes.hpp:15

M\_

int M\_

**Definition:** mdivide\_left.hpp:19


---

[ Stan Home Page ]

*© 2011–2014,
Stan Development Team.*
